# Diabetes in the workplace - diabetic’s perceptions and experiences of managing their disease at work: a qualitative study

**DOI:** 10.1186/1471-2458-13-386

**Published:** 2013-04-25

**Authors:** Annmarie Ruston, Alison Smith, Bernard Fernando

**Affiliations:** 1 Centre for Health and Social Care Research, Faculty of Health and Social Care, Canterbury Christ Church University, Medway Campus, Cathedral Court, 30 Pembroke Court, Chatham Maritime, Kent, ME4 4UF, UK; 2 2 Thames Ave, Rainham, Gillingham, Kent, ME8 9BW, UK

**Keywords:** Diabetes, Workplace, Disease management

## Abstract

**Background:**

Diabetes represents one of the biggest public health challenges facing the UK. It is also associated with increasing costs to the economy due to working days lost as people with diabetes have a sickness absence rate 2–3 times greater than the general population. Workplaces have the potential to support or hinder self- management of diabetes but little research has been undertaken to examine the relationship between work and diabetes in the UK. This paper seeks to go some way to addressing this gap by exploring the perceptions and experiences of employees with diabetes.

**Methods:**

Forty three people with diabetes were purposively recruited to ascertain ways in which they managed their disease in the workplace. Semi-structured, interviews were undertaken, tape recorded and transcribed. Analysis was conducted using a constant comparative approach.

**Results:**

Although respondents had informed managers of their diabetic status they felt that their managers had little concept of the effects of the work environment on their ability to manage their disease. They did not expect support from their managers and were concerned about being stigmatised or treated inappropriately. Work requirements took priority. They had to adapt their disease management to fit their job and reported running their blood glucose levels at higher than optimal levels, thereby putting themselves at higher risk of long term complications.

**Conclusions:**

Little research has examined the way in which employees with diabetes manage their disease in the workplace. This research shows there is a need to increase the awareness of managers of the short and long term economic benefit of supporting employees with diabetes to manage their disease effectively whist at work. Employees may need individually assessed and tailored support on the job in order to manage their disease effectively.

## Background

### Diabetes

Diabetes represents one of the biggest public health challenges facing the UK [1]. It is estimated that around 3.8 million people the UK are living with diabetes and by 2035 it is expected to rise to 6.25 million with a increasing proportion of cases arising in the working age population [2,3]. Approximately, 10% of adults with diabetes have type 1 diabetes which typically develops before the age of 40, is neither preventable nor curable and is treated with insulin either by injection or pump, a healthy diet and regular physical activity [1]. Rigid control of lifestyle risk factors and strict glycemic control are needed to manage type I diabetes [4]. Type 2 diabetes usually occurs in people aged over 40, it is a largely preventable, non-curable disease which is treated with diet and regular physical activity, but medication and/or insulin may be required [4]. Approximately, 90% of adults with diabetes have this type of diabetes [1].

Diabetes is associated with serious complications including heart disease, stroke, blindness, kidney disease, nerve damage, amputations leading to disability and premature mortality [1]. These complications potentially influence an individual’s ability to do their job effectively and result in costs to the economy due to working days lost [5]. Indeed people with diabetes have a sickness absence rate 2–3 times greater than the general population [5,6]. They are 10 – 20 times more likely to go blind than those without the disease [7,8] and damage to the nerves in feet also results in approximately 100 people per week losing a toe, foot or lower limb due to diabetes [1]. People with diabetes have a five fold increased risk of developing cardiovascular disease, are five times more likely to suffer heart failure and at least 15% of deaths in those with type 2 diabetes is the result of a stroke [1].

Evidence demonstrates that strict glycemic control and rigid control of lifestyle factors can significantly reduce the risk of developing these long term complications [4,9] but that this is not necessarily being effectively realised with many people with diabetes failing to achieve optimal outcomes and experiencing devastating complications that result in a decreased length and quality of life [10]. Funnell et al. [10] attribute this to the fact that the effort of healthcare professionals has traditionally been spent on developing methods for ensuring compliance with prescribed therapeutic regimens rather than understanding the complexity and reality of managing diabetes on a daily basis [10]. It is estimated that 95% of diabetes management is self-management which requires people with diabetes to make multiple daily self-care decisions within the context and constraints of their everyday lives, including their time at work [1].

Given the rise in diabetes amongst working populations and the length of time most people spend at work, the workplace has considerable potential to influence the way in which employees manage their diabetes. However, evidence suggests that for many people with diabetes the workplace, rather than being health promoting presents a number of challenges. These include the choice of work, relationships with colleagues and managers and disease management issues such as difficulties in insulin administration, blood glucose monitoring and difficulties in securing time off to attend appointments [11,12]. Additionally, evidence from the international literature about the implementation of policies on work adjustment, work-life balance and health and safety have demonstrated that effectiveness of such policies for diabetes has been mixed [13-15].

There is a dearth of literature which examines the way in which employees with diabetes manage their disease while at work and this paper addresses this gap.

### Aim

The aim of this paper is to examine the ways in which people with type 1 and type 2 diabetes accessed support for and managed their diabetes whilst at work and to identify factors that presented barriers to effective management.

## Methods

The data presented in this paper are drawn from a qualitative study to gain an understanding of how people with diabetes, living in England, accessed support for managing their disease within the workplace and from health services, their perceptions of the support and how this support influenced their self management of the disease. The research approach used was phenomenology which accepts experience as it exists in the consciousness of the individual [16].

### Study design and sample

The inclusion criteria for the study were current or recent employment and mode of treatment. People with type 1 diabetes need to administer insulin either through daily injections or through an insulin pump and generally attend specialist centres, only some of which support the use of insulin pumps. To ensure inclusion of those using insulin pumps and multiple injections the study utilised a national database, already held within the University, of people with type 1 diabetes. The database was compiled from users of an online support organisation which advocates access to insulin pumps and other diabetes technologies in the UK [17]. People with type 2 diabetes were recruited via local diabetes clinics in two general practices in south east England.

Recruitment of both those with type 1 and type 2 diabetes continued until data saturation was reached. A total of 43 respondents were interviewed 23 females and 20 males. Thirty two had type 1 diabetes, of these 23 currently used insulin pump therapy and 9 used multiple daily injections. The remaining 11 respondents, 8 male and 3 female, had type 2 diabetes and used a combination of diet, exercise and medication to manage their disease. The majority, 44% (19), of respondents worked in the private sector, 37% (16) in the public sector, 7% (3) in the voluntary sector and 12% (5) had moved into self employment due to their diabetes. The sample characteristics according to employment sector are presented in Table 1.

**Table 1:** Sample characteristics by employment sector

**Employment Sector**	**Sex**	**Age**	**Type of diabetes**	**Current treatment**	**Occupation**
Public Sector	F	45	Type 1	Injections	Social Worker- Local Government
Public Sector	F	41	Type 1	Pump	Health Professional
Public Sector	F	58	Type 1	Pump	Health Professional
Public Sector	F	48	Type 1	Pump	Health Professional
Public Sector	F	64	Type 1	Pump	Lecturer Education Sector
Public Sector	M	45	Type 1	Pump	Environmental Health Officer
Public Sector	F	62	Type 1	Injections	Health Professional
Public Sector	F	54	Type 1	Injections	Librarian
Public Sector	F	53	Type 1	Pump	Health Professional
Public Sector	F	64	Type 1	Pump	Health Worker
Public Sector	F	53	Type 1	Pump	Design co-ordinator Local Authority
Public Sector	M	54	Type 1	Injection	Civil Servant- Public Administration
Public Sector	F	62	Type 2	Medication/diet	Teacher – Education sector
Public Sector	F	54	Type 2	Medication/diet	Health Professional
Public Sector	M	61	Type 2	Medication/diet	Lecturer
Public Sector	F	63	Type 2	Medication/diet	Teacher
Self employed	F	49	Type 1	Pump	Writer
Self employed	F	55	Type 1	Pump	Gardner
Self employed	M	46	Type 1	Pump	Editor/typesetter
Self employed	F	44	Type 1	Pump	Secretarial services
Self employed	M	60	Type 2	Medication/diet	Service Sector
Voluntary Sector	M	65	Type 1	Pump	Co-ordinator
Voluntary Sector	F	33	Type 1	Pump	Administrator – Social Care Sector
Voluntary Sector	M	60	Type 1	Injections	Charity Worker
Private Sector	M	44	Type 1	Injections	Manager Transport Sector
Private Sector	M	59	Type 1	Injections	Accountant – Financial Sector
Private Sector	M	62	Type 1	Pump	Company Director
Private Sector	M	33	Type 1	Injections	Food industry
Private Sector	M	53	Type 1	Pump	Accountancy
Private Sector	F	39	Type 1	Pump	Customer Services Transport Sector
Private Sector	F	30	Type 1	Pump	Check in Assistant - Transport Sector
Private Sector	F	50	Type 1	Pump	Sales Assistant – Retail Sector
Private sector	M	65	Type 1	Pump	Salesman Retail Sector
Private Sector	F	65	Type 1	Pump	Receptionist – Complementary Health
Private Sector	F	40	Type 1	Pump	Office Worker – Service Sector
Private Sector	F	50	Type 1	Pump	Learning support – Education Sector
Private Sector	M	45	Type 1	Injection	Sales – Retail Sector
Private Sector	M	55	Type 2	Medication/diet	Accountancy
Private Sector	M	60	Type 2	Medication/diet	Finance officer
Private Sector	M	47	Type 2	Medication/diet	Human Resources – Local Government
Private Sector	M	50	Type 2	Medication/diet	Packer – Manufacturing Sector
Private Sector	M	59	Type 2	Medication/diet	Manager – Leisure Sector
Private Sector	M	60	Type 2	Medication/diet	Engineer – Construction Sector

### Data collection

People with type 1 diabetes were approached by telephone and their employment status and treatment mode ascertained, the study was explained to them and a suitable time agreed for interview. Telephone interviews were conducted due to the geographical spread of the sample.

People with type 2 diabetes were recruited by the researcher sitting in on diabetes clinics and approaching potential participants. Those meeting the inclusion criteria, were provided with information about the study and asked to take part at their convenience and were interviewed face to face either in the GP practice or in a setting of their choice. The interviews were semi-structured and covered participants perceptions of the role of health professionals, managers and work colleagues in supporting and managing their disease, who they considered to be responsible for the management of the disease, the barriers they encountered in self management, factors that facilitated good management and what could be done to support them.

The study gained local NHS Research Ethics clearance (07/076P 444) for access to NHS patients and University ethics approval for access to patients from the database.

### Data analysis

The interviews, lasted between 30 and 40 minutes, were tape recorded and transcribed. The literature review had supported the development of the interview schedule, however, preliminary analysis of data was undertaken concurrently with data collection to identify and incorporate emerging themes and ensure that sufficient respondents were recruited to achieve data saturation in relation to their to access to support for their diabetes in both in the workplace and the health services. Initial analysis was conducted using a constant comparative approach [18]. Each transcript was separately read by two members of the research team (AR and AS) to identify emerging themes. As new themes emerged they were incorporated into the interview guide and addressed in further interviews. Emerging themes relating to employment included type of work, reaction of employers and colleagues to condition, ability to carry out job, management of diabetes at work and effect of work on the disease. The transcripts were re-read and themes compared with one another to identify similarities and differences. The themes were then refined to ensure that the concepts, relations between variables and differences between the themes could be confirmed or modified as necessary. Following the initial analysis further exploration of relationships and patterns across themes was undertaken in relation to type of diabetes. To ensure trustworthiness of the research the researchers own beliefs and pre-conceptions were suspended (bracketed) during interviewing and data analysis. In addition the third member of the research team acted as a ‘peer debriefer’ ensuring that the results were grounded in respondents reports [18].

## Results

Moser et al. [19] argued that diabetes self-management is a lifelong matter which takes shape through individuals establishing their own personal self-management strategies. In describing their diabetes self management strategies respondents reported that they considered themselves to be ‘experts’ in their own disease and its management. Type 1 respondents, having experienced the disease for a long period of their lives reported highly developed skills aimed at managing their diabetes:

“I’ve sort of invented my own ways of dealing with it (diabetes) ..which works for me and I prefer to control it myself.” Int 14, Type 1, Check in Assistant, Transport Sector

Type 1 respondents generally reported a desire to maintain control of their disease themselves rather than rely on others, whilst type 2 respondents reported being more willing to comply with guidance from their health professionals.

In this context most respondents considered that it was their own responsibility to manage their disease while at work and to ensure that they had what they needed to maintain appropriate blood glucose levels. Respondents considered that it was not appropriate to expect their workplace or company to take any form of ownership or responsibility for their disease:

“*I think that, you know, you’ve got to erm, take, the individual’s got to take responsibility for it I think and er, you know, er and really manage themselves, you know and er, I don’t think it should come in on the company sort of thing, you know make sure that you’re bringing in your little packed lunch or the right lunch… you have to take responsibility yourself really” Int. 42, Type 2, Manager, Leisure Sector.*

However, this sense of personal responsibility and need for personal control appeared to have important implications for the wellbeing of respondents while at work. Two key themes emerged from the data. Firstly, that respondent’s did not see any real value in informing their employers about their diabetes, nor did they expect much support from them. Secondly, they reported running their blood glucose levels at higher than optimal levels to manage their work, thereby putting themselves at a greater risk of developing long term complications.

### Informing employers of their diabetes status and gaining support

Managing chronic illness involves the adoption of a styles of adjustment which include deciding how much should be disclosed or disguised about the condition, how far the person should ‘come out’ and in what way and in interacting with others [20,21].

Munir et al. [22] identified a range of factors that may influence whether an employee will self disclose his or her illness including the need to take medication at work, sickness absence, the impact of the illness on their ability to do the work, access to practical or social support, possible stigma associated with the illness and the organisational culture. Self disclosure can be either full or partial. Partial disclosure is where the employee does no more than inform their line manager about the presence of an illness:

“When I was diagnosed as diabetic I advised them, but that was more from the point of view that a) they needed to know b) er because I drive a company car, they needed to know to advise insurers – but I don’t think to be honest with you that it registers on their radar screen so much that that I’m a diabetic.” Int 43 Type 2, Engineer, Construction Sector.

Full self disclosure, which involves employees informing their line managers about how their illness affects them whilst they are at work, has been shown to be more likely to occur where employees perceive they will receive support from their line managers [22]. In our study full disclosure of diabetes status, whether desired or not, often followed a hyperglycaemic (increased thirst, increased urination, tiredness or fatigue and blurred vision) or hypoglycaemic (dizziness, shaking, slowed speech or thinking and weakness and possibly mental confusion, unconsciousness or seizures) event.

Of the 19 respondents who worked in the private sector 5 were forced to disclose their diabetes due to having had a hypoglycaemic event or time off sick. They reported their line managers as unhelpful and received little support. Six reported voluntary full disclosure and considered that they had been given a good level of support within their workplace. While the remaining 8 respondents reported partial disclosure only 1 of whom reported receiving support.

Of those working in the public or voluntary sector (19) diabetes status was disclosed as a result of them having had a hypoglycaemic event in 9 cases and all reported their line managers as unhelpful and unsupportive. Four reported fully and voluntarily disclosing their diabetes and 3 of these reported they had gained support. Six reported partial self disclosure only 1 of whom received any support.

There was little difference between those with type 1 and type 2 diabetes in terms of the support received, however, respondents who were using multiple daily injections and were likely to require special consideration at work due to the need to be able to safely inject themselves, were more likely to have fully disclosed their diabetes and to have gained support. Full voluntary disclosure was more closely linked to gaining support from managers with 9 out of the 10 respondents who voluntarily disclosed their diabetes reporting having gained support. Nevertheless, most respondents (27) reported finding their managers unhelpful and two potential reasons for this lack of support were suggested by respondents.

Firstly, there was a general view that employers and managers did not understand diabetes and therefore were not in a position to provide appropriate support:

“I wouldn’t say they (management) particularly understand, erm, I told them and it’s accepted – but I’m not sure my line manager is capable of understanding.” No 38 Type 2, Human Resources Local Government, Public Administration Sector

The majority of respondents indicated that their managers were unaware of the nature of diabetes or its potential effect on their health and productivity. Linked to this lack of understanding of diabetes was a tendency for managers to be disinterested and therefore not likely to ascertain the level of support that might be needed:

“They know I’m diabetic, but that’s it, they never asked anything about it or what to do.” Int 6, Type 1, Accountant, Financial Sector

Overall, only 9 of the 43 respondents considered that they had had any relevant support from their managers. Those who voluntarily disclosed their diabetes status and reported receiving support stated that they worked in environments where there was a supportive ethos in which managers valued their staff.

Secondly, respondents considered that managers were more concerned about getting the job done than considering the well being of the employee or providing any concessions for their diabetes. Respondents described a number of circumstances in which they felt they were denied the opportunity to undertake activities that were needed to effectively manage their diabetes. For example, the following respondent described how his manager was unhappy about him taking time off work to go to the hospital or doctor:

“At work I get lots of ‘well why do you need to go now (to doctor or hospital) can’t you do it after work, why can’t you do it on your day off (Sunday) ” Int 22, Type 1, Salesman Retail Sector

Difficulty in securing time off for medical appointments, regular meal breaks and safe hygienic areas to administer insulin were identified as problematic.

In addition to managers and employers being perceived as having a poor understanding of diabetes respondents suggested that organisational risk management policies and practices were also problematic and unhelpful. For example, the following respondent echoing the concerns of other respondents, said that their manager tended to over react to any situation where the employee with diabetes felt unwell:

“I’m not sure they (management) fully understand. I think at a drop of a hat they’d phone for an ambulance when I’d really think it wasn’t necessary. I work for a County Council and their sort of Health and Safety policy is that if somebody is unwell at work, then you can’t move them. You must get an ambulance and get them out of the building. That isn’t really how you should treat a diabetic. I think because the policy has been put in place they don’t treat it on an individual basis. Int 30 Type 1, Design Co-ordinator, Local Government Public Administration Sector.

Respondents described health and safety policies and practices as being generic and as consequence not necessarily being suitable for people with diabetes. Thus, rather than managers ascertaining the needs of employees with diabetes and devising a plan or strategy to address those needs managers tended to implement a blanket health and safety policies which involved sending employees with diabetes off to hospital.

Over reliance on health and safety policies were also implicated in respondent’s views about feeling unsupported. For example, the effect of having had a hypoglycaemic event was described by those with type 1 diabetes as distressing and potentially having long term consequences for them while at work:

“I had a ‘hypo’ and my manager decided that I should have a risk assessment. And it was very difficult for me because, from a social work point of view, we do risk assessments on clients. It was extremely humiliating ..it wasn’t occupational health, they didn’t even get involved. It was a manager from another department – she’s not even health trained or anything and they suggested that I have someone with me all through the day in case my diabetes was messed up.” Int no 1 Type 1, Social Worker Local Government, Public Administration Sector

The example above illustrated the potential difficulties employees may face in a workplace where there is a lack of understanding of the nature of the disease. Although the manager addressed the hypoglycaemic event according to protocol it still left the employee feeling unsupported and potentially stigmatised.

Nor did respondents expect that their colleagues would be in a position to do much to help. Most stated that they informed their immediate colleagues of their diabetic status but felt that colleagues, like the managers, did not understand the nature of diabetes and held a number of misconceptions about appropriate preventative behaviours for diabetes:

“I went to work after a hypo and they (colleagues) said ‘Cor, you look rubbish’, ‘Cor you look ill have you been drinking?’ and you just want to be left alone to get back to normal. People who don’t understand, umm, I find come up with statements like ooh you shouldn’t be eating that should you? cos it’s got sugar in.” In 32, Type 1, Salesperson, Retail Sector

A number of respondents (10) also reported experiencing a degree of prejudice from colleagues which added to the constraints they faced in managing their diabetes at work:

“I worked with a very bigoted woman who didn’t want me to do my pen injections at my desk… She wanted me to go into the toilets and do it and I fought my corner and said ‘Actually, no – a sterile piece of medical equipment that I’m trying to put into me, in a really stinky, unhygienic toilet?” Int 8, Type 1 Administrator, Social Care Sector.

Respondents suggested that it took time to be able to educate their colleagues and to trust that they would not just ‘press the button and call an ambulance’ rather than give them some sugary drinks or something to eat as required. Nevertheless, those respondents who worked in smaller workplaces, staffed mainly by females, were more likely to report that they were able to ‘educate’ their colleagues about diabetes and to get them to keep an eye out for an impending ‘hypo’ and provide sugary drinks/food as appropriate.

### Running blood glucose levels above optimum levels

Chronic illness and its outcomes are shaped by the decisions and actions carried out by individuals over of the ‘trajectory’ of the illness [20]. Diabetes is a chronic illness which interferes with social interaction and role performance. Within the workplace it is not just a given biological entity, patterned by social conditions, but is itself a ‘negotiated reality’ [20]. As respondents generally felt unable to negotiate and obtain appropriate support for their illness they reported taking actions to ensure that their diabetes did not limit their ability to carry out their work. They reported ‘controlling’ or managing their disease in a way that would reduce any potential disruption in the workplace. Tight control of blood glucose levels is the most important preventative measure to reduce the risk of long term complications of diabetes and the risk of experiencing a hypoglycaemic event - particularly for those with type 1 diabetes. In order to achieve the goal of controlling their blood glucose levels some respondents reported frequent checking of their blood glucose levels and then adjusting insulin doses or food in order to manage fluctuations in blood glucose levels:

“I review my basal rate (continuous rate of insulin) or I reduce my bolus (the amount of insulin given for food) one or the other sometimes both. I do it for hours on the trot.” Int 27 Type 1 Office worker, Service Sector

However, nearly three quarters (30) of respondents adopted a diabetes management strategy that potentially put their long term health and future productivity at risk. They reported running their blood glucose levels ‘high’ in order to be able to function effectively at work. Central to running blood glucose at higher than optimal levels were three main issues:

Firstly, the need to feel well enough to carry out their work and stay focused. For example, the following respondent, in describing how she managed her diabetes at work, reported that to feel right and be able to do her job she needed to go against advice recommended by her health professionals:

“I think personally in myself in my body I feel better when I’ve go more sugar and when I’m sort of 5 or 6 mmol/l like they (health professionals) want I feel a little bit not right.” Int 20, Type 1, Sales Assistant, Retail Sector

Secondly, for respondents with type 1 diabetes the desire to avoid a hypoglycaemic event while at work was reported as a priority:

“ If anything at work I tend to run slightly higher so that I don’t have hypos” Int 5 Type 1, Manager, Transport Sector

The need for some employees to protect the people they were working with, such as young children or vulnerable adults, provided a rationale for them to run their blood glucose levels at higher than optimal levels in order to avoid a ‘hypo’ occurring at an inappropriate time.

“If I am teaching I tend to run slightly higher because I don’t want to go ‘hypo’ in front of a class – so I tend to run higher if I am in that sort of situation.” Int 7, Type 1, Health Professional, Health Sector

Thirdly, the need to accommodate situations where work patterns meant that they could not access food, monitor their blood sugar etc. which might in turn result in an adverse event:

“I run it higher when I know I will have to go without food at work.” Int 39, Type 2, Packer, Manufacturing Sector

Where respondents worked in situations where there was a requirement to complete tasks within set times, e.g. in a factory production line, and there was no opportunity for the employee to take a break when needed, they were more likely to report running their blood glucose levels high. This was also relevant to employees who undertook tasks that required high levels of concentration and where lives may be put at risk if a ‘hypo’ were to occur:

“I always tended to run on higher blood sugar. And I do a lot of driving, so I could never afford to take the risk of having a ‘hypo’ on the motorway.” Int. 22 Type 1, Salesman, Retail Sector

Even where working conditions were potentially hazardous, rather than alert employers or managers to the potential risks or dangers, respondents preferred to control the situation themselves by allowing higher than optimal levels of blood glucose in order to avoid the panic created by hypoglycaemic events:

“I work in a prison – I work with murders and rapists etc. and er. I work alone with them and I can’t afford to have a ‘hypo’ but then again I don’t want to be as high as I sometimes am. I don’t get the opportunity of doing as many blood tests as I would like because I can’t carry things with me. I can take biscuits and sweets but the needles and medicine etc. has to be locked away. To be honest I would not press the panic button (if felt having a hypo) because it is all about saving face. You don’t want it to infringe on your life to that extent also people over react – so you feel a responsibility about panicking them – so I run high” Int 19 Type 1, Librarian, Public Administration Sector.

Respondents in this study made decisions which resulted in work requirements taking priority over their individual needs and their diabetes self management was adjusted to fit the job rather than the job being re-structured or adapted to meet their needs. Respondents reported adopting strategies that focused on minimising visible loss of productivity e.g. as a consequence of ‘hypo’s’ or not being fit to drive a vehicle etc.

Type 1 respondents tended to report running their blood glucose high in order to prevent the risk of a ‘hypo’ rather than on keeping their blood glucose levels tightly controlled. While type 2 respondents reported trying to ensure they were able to eat food regularly in order to maintain their blood glucose at optimum levels but having to run their blood glucose high in certain circumstances. These strategies reflected a need to ‘manage’ their diabetes in order to reduce its impact at work and to enable them to work independently without support from their employers. Bury [21] suggested that people that are suffering from chronic illness make choices about how to mobilise resources and balance demands on others while remaining independent. Respondent’s in this study, unable to access adequate support and believing that they had responsibility for managing their own disease, resorted to engaging in self management strategies that were likely to be detrimental to their health and independence in the long term.

## Discussion

Diabetes presents a considerable health and economic burden for patients, families, industry and society [23]. Several studies have shown negative associations between diabetes and employment with diabetes affecting employment in a number of ways including absenteeism, impaired productivity and inability to work due to complications of the disease [23]. Within the UK diabetes is associated with increasing cost to the economy due to working days lost. For example, approximately, 5,960,000 working days were lost due to type 2 diabetes in 1998 [5]. As diabetes becomes more prevalent in the working population its negative effect on employment and work productivity are likely to become more pressing [23]. Given this there is a strong rationale for employers to support those in their workforce with diabetes to effectively manage their disease and risk factors.

However, diabetes is largely a self managed disease and successful management of the disease within the workplace would also be dependent on the preventative and help seeking behaviour of those with the disease. There is, however, a paucity of research which has explored the perceptions and experiences of employees in their efforts to manage their diabetes in the workplace. The study reported in this paper has gone some way to addressing this gap in the literature.

Two key findings from the study are of importance. Firstly, that the complexities of diabetes are not fully understood within many workplaces with employers and managers having little concept of the implications of the disease for their employee or of the effects of the work environment on the employee’s ability to manage their disease. Secondly, the study has illuminated the way in which workplace requirements can influence decision making and practices adopted by employees with diabetes. In the case of our respondents this resulted in them running high blood glucose to avoid hypoglycaemia.

There are a number of interrelated implications of this. Firstly, due to limited understanding managers were not able to adequately provide preventative support for their employees to ensure appropriate self management thereby increasing the risk of employees developing diabetes related long term complications. At the same time employees did not expect support from their managers and were reluctant to disclose their illness and raise support issues in case they were stigmatised or treated inappropriately e.g. over reaction to ‘hypos’. The consequence of this action is that employers and managers remained ignorant of the needs of employees.

In the absence of support from employers or managers employees believed that they needed to focus on productivity and doing their job, they managed their diabetes in a way which minimised visibility and disruption to work. One of the main strategies identified being to run their blood glucose levels higher than optimum.

The potential consequences of this sequence of events or practices for organisations and individuals with diabetes are significant. Poor glycaemic control is closely linked to the development of diabetes related complications such as blindness, limb amputations, coronary heart disease and stroke. Thus, individual employees adopting the practices reported in this study were potentially placing not only their future health at risk but also their long term viability as an employee. Whilst, organisations in ignoring the needs of employees with diabetes were likely to experience an increase in absenteeism and loss of productivity from these employees in the longer term. Organisations were also likely to be in breach of the Equality Act 2010 which requires employers to make reasonable adjustments to accommodate employees’ health problems [24].

The findings of our study support others that have suggested that in order to provide effective preventative initiatives and support for empolyees who have diabetes employers and managers need to recognise the importance of the disease, encourage people to disclose their illness and provide a supportive environment in which both individual and organisational constraints can be addressed [1,25].

Given the increasing number of people with diabetes who are part of the workforce there is a need to focus on work based health management strategies and on ensuring that health professionals recognise the potential constraints faced by people with diabetes at work. Implementing workplace support and prevention for people with diabetes will require multi-modal interventions which combine individual health practices, personal resources and the workplace environment, including the organisation’s philosophy [26].

Our findings have added to the literature by illustrating ways in which people with diabetes contribute to the problems associated with managing diabetes at work. The findings suggest that there is a need to educate and support people with diabetes to disclose their illness and actively seek support within the workplace. Our respondents argued that they were responsible for managing their disease and as such they could have been more assertive within the workplace and requested support.

The findings of the study also have implications for the National Health Service as it will ultimately have to absorb the costs associated with treating the complications of diabetes that arise from poor glycaemic control. Most people with diabetes have dedicated health professionals who monitor their disease and there is the potential for these health professionals to support their patients in their negotiations with their workplace about their diabetes.

### Limitations

Qualitative studies in the field of health and illness have provided valuable insights into lay concepts of health maintenance and risk behaviour, however, the applicability of qualitative research findings beyond the sample is open to debate. Nevertheless, they can reveal richness and complexity of people’s beliefs and behaviours. The sample of people with type 1 diabetes, in this study, were derived from a database of people who access online support for their diabetes and this may represent a selection bias. Nevertheless, the principles of purposeful sampling were followed to ensure that data saturation was achieved on the main aspects of the study and the sampling resulted in a reasonable range of occupations being covered.

## Conclusions

Diabetes is one of the biggest public health challenges facing the UK. It has significant implications for the economy and the workplace due to increased levels of sickness absence in this segment of the workforce and its association with a range of disabling complications which may influence an individual’s ability to do their job effectively. This study has highlighted ways in which workplace policies, practices and cultures can influence the individual’s management of their diabetes and the potential for this to be detrimental to their health in the longer term. Workplaces have the potential to support or hinder diabetes self management future efforts in the secondary prevention of diabetes might usefully focus establishing workplace health promotion/management programmes.

## Competing interests

The authors declare that they have no competing interests in the conduct of this study.

## Authors’ contributions

AR developed the protocol, AS undertook data collection, AR and AS undertook the analysis. BF contributed to the study design and to the interpretation of the findings. All authors contributed to the manuscript drafts and approved the final manuscript.

## Pre-publication history

The pre-publication history for this paper can be accessed here:

http://www.biomedcentral.com/1471-2458/13/386/prepub
